# Recalculation of inhibitor concentration fold-changes for CMY-219: comments on a recent article about CMY-219 conferring resistance to ceftazidime-avibactam

**DOI:** 10.1128/aac.00505-26

**Published:** 2026-06-24

**Authors:** Chunlin Feng

**Affiliations:** 1 Department of Laboratory Medicine, The Fourth Affiliated Hospital of Southwest Medical University, Meishan, China; University of Pennsylvania Perelman School of Medicine, Philadelphia, Pennsylvania, USA

**Keywords:** CMY-219, beta-lactamase, CMY variants

## LETTER

We read with great interest the recent report by Jousset et al. in *Antimicrobial Agents and Chemotherapy* on 12 February 2026 (1). While the study provides a valuable susceptibility profile, there are several reporting issues that warrant discussion to ensure the precision and reproducibility of these biological findings.

The authors stated that the avibactam IC_50_ values were “27-fold higher” for CMY-219 compared to CMY-42, and the cloxacillin IC_50_ values were “4,000-fold” increased. For avibactam, the reported IC_50_ for CMY-219 was 9,900 nM, while the IC_50_ for CMY-42 was 400 nM. Dividing 9,900 by 400 yields a factor of 24.75, rather than the published 27-fold increase. The IC_50_ for CMY-219 was reported as 22,000 nM, and the IC_50_ for CMY-42 was 3.7 nM. Comparing the variant yields an increase of approximately 5,946-fold.

The finding that CMY-219 exhibited profound resistance to ceftazidime-avibactam was clinically alarming. However, accurate data are critical for downstream applications such as molecular docking. We would appreciate it if the authors could clarify the methodology behind the reported ratios.
